# Interaction of myelin basic protein with cytoskeletal and signaling proteins in cultured primary oligodendrocytes and N19 oligodendroglial cells

**DOI:** 10.1186/1756-0500-7-387

**Published:** 2014-06-24

**Authors:** Joan M Boggs, Lopamudra Homchaudhuri, Godha Ranagaraj, Yuanfang Liu, Graham ST Smith, George Harauz

**Affiliations:** 1 Molecular Structure and Function Program, Research Institute, Hospital for Sick Children, 686 Bay St, Toronto, ON M5G 0A4, Canada; 2 Department of Laboratory Medicine and Pathobiology, University of Toronto, Toronto, ON, Canada; 3 Department of Molecular and Cellular Biology, University of Guelph, 50 Stone Road East, Guelph, ON N1G 2W1, Canada; 4 Present address: Neurosciences and Mental Health Program, Research Institute, Hospital for Sick Children, Toronto, ON, Canada

**Keywords:** Oligodendrocyte, Myelin basic protein, Actin, Tubulin, ZO-1, Cortactin, Fyn kinase, Co-immunoprecipitation, Confocal microscopy, Live-cell imaging

## Abstract

**Background:**

The classic myelin basic protein (MBP) isoforms are intrinsically-disordered proteins of 14–21.5 kDa in size arising from the *Golli* (Gene in the Oligodendrocyte Lineage) gene complex, and are responsible for formation of the multilayered myelin sheath in the central nervous system. The predominant membrane-associated isoform of MBP is not simply a structural component of compact myelin but is highly post-translationally modified and multi-functional, having interactions with numerous proteins such as Ca^2+^-calmodulin, and with actin, tubulin, and proteins with SH3-domains, which it can tether to a lipid membrane *in vitro*. It co-localizes with such proteins in primary oligodendrocytes (OLGs) and in early developmental N19-OLGs transfected with fluorescently-tagged MBP.

**Results:**

To provide further evidence for MBP associations with these proteins *in vivo*, we show here that MBP isoforms are co-immunoprecipitated from detergent extracts of primary OLGs together with actin, tubulin, zonula occludens 1 (ZO-1), cortactin, and Fyn kinase. We also carry out live-cell imaging of N19-OLGs co-transfected with fluorescent MBP and actin, and show that when actin filaments re-assemble after recovery from cytochalasin D treatment, MBP and actin are rapidly enriched and co-localized at certain sites at the plasma membrane and in newly-formed membrane ruffles. The MBP and actin distributions change similarly with time, suggesting a specific and dynamic association.

**Conclusions:**

These results provide more direct evidence for association of the predominant 18.5-kDa MBP isoform with these proteins in primary OLGs and in live cells than previously could be inferred from co-localization observations. This study supports further a role for classic MBP isoforms in protein-protein interactions during membrane and cytoskeletal extension and remodeling in OLGs.

## Background

Myelin basic protein (MBP, specifically the classic membrane-targeted 18.5-kDa isoform) is responsible for adhesion of the cytoplasmic surfaces of the multilayered myelin sheath [1,2], and may form a molecular sieve restricting many oligodendroglial proteins from access to compact myelin [3-6]. It is an intrinsically-disordered protein that acquires local elements of ordered structure on binding to lipids or to other proteins [7-15]. Like other intrinsically-disordered proteins, it binds to many other proteins *in vitro*, both through electrostatic interactions and through a PXRP SH3-ligand domain [15,16]. Those protein-protein interactions that have been studied in detail include actin, tubulin, Ca^2+^-calmodulin, and the SH3-domain proteins Fyn-kinase, ZO-1, and cortactin [10,11,15-23]. Furthermore, MBP can polymerize and bundle actin filaments and microtubules, cross-link them to each other, and tether them and SH3-domain proteins (as demonstrated for the SH3-domain of Fyn-kinase) to a lipid surface [24-26]. These varied interactions may allow MBP to participate as a signaling hub in myelin formation and remodeling, and thus to have many other functions in addition to membrane adhesion in compact multilamellar myelin [11,16,27]. *In vitro*, Ca^2+^-calmodulin dissociates MBP from these proteins and from the lipid bilayer, and thus could regulate this signaling role *in vivo*[24,28]. Post-translational modifications to MBP, such as phosphorylation and deimination, which reduce its net positive charge, and increased membrane surface charge due to increased amounts of negatively-charged lipids, can also regulate these interactions, and may be important modulators of myelin assembly and turnover [14,17,25,29-33].

These diverse associations with MBP have been characterized extensively using purified proteins and lipid vesicles *in vitro*. Concomitantly, co-localization of MBP and cytoskeletal and SH3-domain proteins has been detected in primary oligodendrocytes [17,25,34-38], and between fluorescent proteins transfected into N19 oligodendroglial cells (N19-OLGs) [27,32,39]. Treatments of cell cultures with PMA (phorbol-12-myristate-13-acetate) or IGF-1 (insulin-like growth factor-1), demonstrated this co-localization more clearly in plasma membrane regions in which cytoskeletal formation was induced, supporting the conclusion that the co-localization is specific and has a physiological role [39]. Classic MBP has also been shown to be important for formation of the cytoskeleton and for stabilizing microtubules in the cold in primary OLGs [40-43]. Beyond such cell-culture systems, MBP has also been co-immunoprecipitated with microtubules from brain tissue [44], and it has been shown by proteomics analysis to be one of many MAPs (microtubule-associated proteins) associated with microtubules from brain [45]. Pull-down assays have also revealed a potential interaction of MBP with β-tubulin and the cytoplasmic loop of the gap junction protein connexin-43 [46].

In this present study, we show first that immunoprecipitation of MBP from primary OLG cell lysates also pulls down cytoskeletal and SH3-domain proteins. Second, we provide further evidence for rapid interaction of MBP with actin filaments formed at certain sites in N19-OLGs after cell recovery from cytochalasin D (CytD) treatment, which directly affects the actin cytoskeleton. These combined results provide more and direct confirmation for specific association of MBP with these proteins in primary OLGs, and in live cells, than previously detected from microscopical co-localization observations. The rapid redistribution or enrichment of MBP at sites of newly formed actin filaments shows that this association is dynamic, and provides further confirmation that this protein plays a physiological role in cytoskeletal remodeling in oligodendroglial cells.

## Methods

### Materials

Mouse monoclonal anti-actin antibody (Clone ACTN05, IgG1), and mouse monoclonal anti-Fyn antibody (p59fyn, clone 1S, IgG1), were purchased from ThermoScientific/LabVision (Fremont, CA); rabbit polyclonal anti-MBP antibody (E13), IgG fraction, was a gift from Dr. E. Day [47]); mouse monoclonal anti-MBP (clone SMI-99), purified IgG2b, was from Covance (Emeryville, CA); mouse monoclonal anti-axotrophin (B2), purified IgG2, and rat monoclonal anti-ZO-1 antibody (R40.76), IgG2a, were from Santa Cruz Biotechnology (Santa Cruz, CA); rabbit polyclonal anti-α/β-tubulin antibody and rabbit polyclonal anti-cortactin antibody were purchased from Cell Signaling Technology (Beverly, MA); and goat anti-rabbit IgG conjugated to HRP (horseradish peroxidase) was purchased from Jackson ImmunoResearch Labs (West Grove, PA). The enhanced chemiluminescence ECL™ Western Blotting reagents were from GE Health Care (Buckinghamshire, UK).

The cross-linking reagent, bis(sulfosuccinimidyl) suberate (BS^3^) was from ThermoScientific (Rockford, IL). Protein G-conjugated Dynabeads were from Life Technologies (Carlsbad, CA). The CytD solution in DMSO and Triton X-100 (SigmaUltra; t-octylphenoxypolyethoxyethanol; TX-100) were purchased from Sigma-Aldrich (St. Louis, MO). The detergent Nonidet® P-40 (Lot 110 F-39211; octylphenoxypolyethoxyethanol) was purchased from Sigma Chemical some time ago, when it was available, and is said to be identical to Igepal, which is still available from Sigma-Aldrich. Both TX-100 and Nonidet® P-40 (NP-40) are from the family octylphenol poly(ethyleneglycolether)_n_, where n is 9.6 for TX-100 and 9.0 for NP-40 [48]. Sodium deoxycholate (99% purity; DOC) was purchased from Bioshop Canada (Burlington, ON). Rhodamine-phalloidin was from Molecular Probes (Eugene, OR).

### Oligodendrocyte culture and cell lysis

Spinal cord oligodendrocytes from Wistar rat 8 day old pups (Charles River Canada, St. Constant, QC) were cultured for 7 days as described previously [49]. They were plated at a cell density of 10^5^/cm^2^ in four-well plates. Four plates of cells were used for each experiment. Culture conditions were identical for all experiments in order to achieve a similar degree of OLG maturation state, with large membrane sheets, and degree of contamination (about 10%) by other cells such as astrocytes. Lysis buffer was 10 mM HEPES-KOH containing 50 mM KCl, 1 mM MgCl_2_, 2 mM EGTA, 2 M glycerol, and 1% TX-100 and 1 mM protease inhibitors (adapted from [37]). Other detergents, 1% NP-40 and 1% sodium deoxycholate (DOC), were added for some experiments, as indicated in the text, to increase the stringency of extraction. The cells were detached and lysed by adding 250 μL of lysis buffer to one plate at room temperature. In order to concentrate and remove as much cell protein as possible, the plate was scraped for 3 min and the suspension transferred to the 2^nd^ plate, which was scraped similarly, and the suspension transferred to the 3^rd^ and 4^th^ plates in turn, and then to a test tube. This step was repeated with another 250 μL of lysis buffer three times. The total volume of 1 mL of suspension from the 4 plates was pooled in one test tube. The sample was incubated for 30 min at room temperature with gentle vortexing every 10 min to complete lysis. Two 5-μL aliquots were removed for protein assay and the sample was frozen overnight. The protein concentration of the pooled material ranged from 2–5 mg protein/mL for different experiments.

### Immunoprecipitation

A flowchart providing an overview of the extraction, immunoprecipitation, and western blotting process is provided in Additional file 1: Figure S1. Lysed cell samples were thawed and centrifuged at 14,000 g for 10 min, the supernatant was removed, and the pellet was resuspended in 1 mL lysis buffer. Aliquots of both the supernatant and resuspended pellet were removed for protein analysis, and both pellet and supernatant were pre-cleared with 150 μL Dynabeads Protein G by rotation for 10 min at room temperature. Each pre-cleared sample (450 μL) was used for immunoprecipitation with monoclonal anti-MBP SMI 99-conjugated or with control (anti-axotrophin) antibody-conjugated Dynabeads.

To reduce the amount of antibody heavy chain and light chain in the immunoprecipitated sample, the monoclonal anti-MBP SMI 99 or control (anti-axotrophin) antibodies were cross-linked to Dynabeads Protein G at a ratio of 25 μg IgG per 150 μL Dynabeads Protein G (4.5 mg) in 800 μL conjugation buffer containing 20 mM sodium phosphate, 150 mM NaCl, at pH 7.4. The beads were rotated for 20 min at room temperature, removed, and washed with 800 μL phosphate-buffered saline (PBS) containing 0.02% Tween 20, and then with 800 μL conjugation buffer. Then 1 mL of 5 mM BS^3^ was added, and the beads were rotated for 30 min at room temperature. Quenching buffer (50 μL, 1 mM Tris–HCl, pH 7.5) was added and the beads were rotated for 15 min. The beads were removed and washed twice with 800 μL lysis buffer.

The pre-cleared lysate pellet and supernatant, both in the original lysis buffer, were added to antibody-conjugated beads and rotated for 2 hours at 4˚C. The beads were removed and washed 6 times with 1 mL lysis buffer by rotation for 5 min. The beads were then extracted to remove bound proteins with 2X NuPage sample buffer (with 4 M urea and 20 mM NaF added) plus an equal volume of distilled water, by boiling for 10 min. The supernatant was removed from the beads, DTT added to it to a final concentration of 5 mM, and the sample boiled again for 10 min. For analysis of MBP, actin, tubulin, and Fyn, aliquots were run on NuPage 4-12% Bis-Tris gels (Life Technologies, Carlsbad, CA) in MES-SDS running buffer. For analysis of ZO-1 and cortactin, NuPAGE 4-12% Bis-Tris gels were used with MOPS-SDS running buffer.

A control antibody was always used in every immunoprecipitation experiment to determine specificity. Significant amounts of proteins of interest were also precipitated by the control antibody-bound beads, probably due to the presence of large agglomerates which may have been membrane-bound. Therefore, the immunoprecipitation experiments were repeated several times with freshly prepared antibody-bound beads for each experiment. Other proteins were concluded to be co-immunoprecipitated with MBP if the anti-MBP-bound beads reproducibly precipitated more of the protein of interest than the control antibody-bound beads.

### Western blotting

Total protein was assayed by the BioRad microassay using Dye Reagent Concentrate from BioRad (Hercules, CA). Proteins were transferred from gels to nitrocellulose membranes using NuPAGE Transfer buffer. The blots were blocked with 5% non-fat skim milk in TBS/0.1% Tween-20. Primary and secondary antibodies were added in the buffer used for blocking the blots. Since antibody light chain and heavy chain had M_*r*_ values close to those of many of the OLG proteins of interest, the following steps were taken to ensure that bands observed on western blots were not light chain or heavy chain: (i) cross-linking of antibody to the Dynabeads Protein G; (ii) use of different animal species of antibody for western blot than the mouse antibodies used for immunoprecipitation, where possible; (iii) use of animal species-specific 2^nd^ antibodies; and (iv) inclusion of the antibodies used for immunoprecipitation on the same gel with OLG samples, in order to determine the location of light chain and heavy chain on the blot. This strategy was used for all gels. Control immunoprecipitates were run on the same gel and used for the same blot as the anti-MBP immunoprecipitate, and band densities were only compared when from the same blot and same exposure time. Western blot procedures were identical for all experiments.

### Plasmid construction

We constructed plasmids coding for RFP-tagged versions of classic 18.5-kDa MBP possessing a 3′UTR (untranslated region) namely, pERFP-C1-rmMBPC1-UTR, as previously described [50] (*n.b.*, the “C1” of the RFP vector designation is not to be confused with the “C1” charge component of MBP). The GFP-tagged β-actin was constructed using recombinant DNA techniques as described previously [39].

### Cell line (N19) culture and transfection

Tissue culture reagents were purchased from Gibco/Invitrogen (Invitrogen Life Technologies, Burlington, ON). The FuGene HD transfection reagent was purchased from Roche (Roche Diagnostics, IN). The N19 immortalized oligodendroglial cell line was grown in high-glucose Dulbecco’s modified Eagle medium (DMEM) supplemented with 10% FBS (foetal bovine serum) and 1% penicillin/streptomycin, and cultured in 10-cm plates at 34°C/5% CO_2_. At 70-80% confluency (4–7 days), the cells were detached using 0.25% trypsin for 5 min, and were seeded onto 2-cm plates containing a glass coverslip. Cells were grown overnight to a confluency of 15% prior to transfection using 100 μL serum-free media, 0.75-3.0 μg of plasmid DNA, and 4 μL of FuGene HD (Roche Diagnostics, IN). The DNA was allowed to complex for 5 min at room temperature, and was directly added to cells following incubation. Cells were cultured for an additional 48 hours at 34°C prior to treatment, fixation, or immunoprocessing.

### N19 cell culture, transfection, and treatment with CytD, live-cell imaging and image analysis

The N19-OLGs were seeded on poly-L-lysine coated coverslips (18-mm diameter, #1.5, Warner Instruments) at 34°C in 5% CO_2,_ as described previously [39]. One day (24 hours) after seeding, the cells were co-transfected with RFP-MBP and GFP-β-actin plasmids respectively. The CytD solution in DMSO was purchased from Sigma-Aldrich at a concentration of 9.9 mM (5 mg/mL) and was diluted in serum-free DMEM (GIBCO) to a final concentration of 2 μM. Two days (48 hours) after transfection, the cells were washed and incubated with fresh serum-free DMEM medium for at least 1.5 hours, and were then treated with 2 μM CytD at 34°C in 5% CO_2_.

In preliminary experiments, the cells were incubated with CytD for varying periods of time, from 30 min to 2 hours, and the cells were fixed and stained with rhodamine-phalloidin [49]. Clumps of actin, rather than long actin filaments were observed after CytD treatment, as observed for OLGs after a 4-hour incubation with CytD [34], and the effect was maximal at 2 hours. The cells were washed 3 times in fresh serum-free DMEM medium (pre-warmed to 34°C) and cultured for additional periods of time up to 2 hours, fixed and stained with rhodamine-phalloidin. Maximal recovery of staining characteristic of actin filaments had occurred after incubation for 1 hour. Therefore, a 2-hour incubation time with CytD, followed by washing and culture for an additional 1-hour recovery time, were used for live cells containing RFP-MBP and GFP-actin. An image of the live cells right after 2-hour culture with CytD was acquired, and CytD was removed by quickly washing the cells 3 times in fresh serum-free DMEM medium (pre-warmed to 34°C). After the last addition of serum-free DMEM medium, acquisition of images was resumed. The N19-OLGs, co-expressing RFP-MBP and GFP-β-actin, were imaged on an Olympus IX81 inverted fluorescence microscope equipped with a Hamamatsu C9100-13 back-thinned EM-CCD camera and a Yokogawa CSU X1 spinning disk confocal scan head. The cells were irradiated with light from separate diode-pumped solid state laser lines at 491 and 561 nm, respectively, and viewed at 60X magnification through a 1.35 N.A. oil-immersion objective. The software used for image acquisition was the Perkin Elmer Software Volocity (Version 5.4.2), and images were processed using the NIH software ImageJ (Version 1.46 g).

Cells were placed on the microscope stage within 2 hours of introducing CytD, and were maintained at 34°C on the microscope stage. This treatment time was chosen as optimal on the basis of control experiments of fixed N19-cells (both transfected with RFP-MBP and untransfected) and staining with phalloidin [51,52]. They were imaged initially at the end of 2 hours exposure to CytD, and every 20 min thereafter for an hour following removal of CytD. Correlation analysis was done by comparing the intensity values due to MBP RFP and β-actin GFP along a line drawn across a region where changes in their distribution were observed after removal of CytD from the cell medium.

## Results

### Immunoprecipitation

Since classic 18.5-kDa MBP is a membrane-associated protein, immunoprecipitation by anti-MBP antibody may bring down membranous agglomerates in which other proteins are present but not directly bound to MBP. Therefore, several detergent buffers of increasing solubilizing ability were used for lysis and immunoprecipitation of primary OLGs. These contained: (i) 1% TX-100; (ii) 1% TX-100 plus 1% NP-40; (iii) 1% TX-100 plus 1% NP-40 plus 1% DOC. These agglomerates may also stick non-specifically to control antibody-bound beads. Therefore, all immunoprecipitation experiments were repeated several times with freshly prepared control antibody and anti-MBP-bound beads to ensure reproducibility.

#### ***Extraction and immunoprecipitation with buffer containing 1% TX-100***

**Figure 1:**
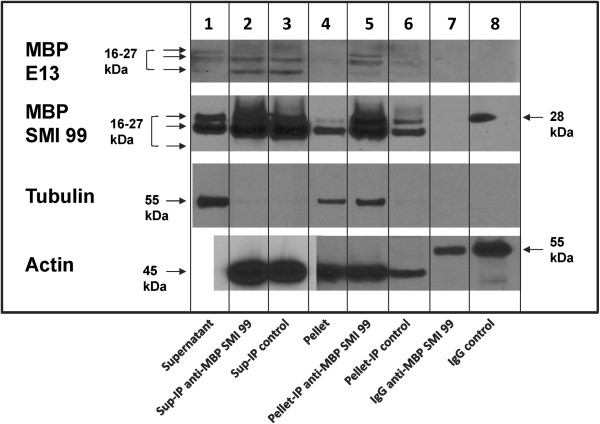
**First level of stringency – extraction with Triton X-100.** MBP, tubulin, and actin co-immunoprecipitate from the OLG Triton X-100 insoluble pellet. Primary OLGs were extracted with buffer containing 1% TX-100, and the soluble and resuspended insoluble fractions were immunoprecipitated in the lysis buffer with monoclonal anti-MBP SMI 99 antibody and a control antibody. Western blots were immunostained with rabbit polyclonal anti-MBP (E13) antibody, mouse monoclonal anti-MBP antibody (SMI 99), rabbit polyclonal anti-tubulin antibody, and mouse monoclonal anti-actin antibody. Representative results of 4 experiments are shown. Lane 1, supernatant fraction; lane 2, anti-MBP (SMI 99) immunoprecipitate of supernatant; lane 3, control antibody immunoprecipitate of supernatant; lane 4, pellet; lane 5, anti-MBP (SMI 99) immunoprecipitate of pellet; lane 6, control antibody immunoprecipitate of pellet; lane 7, anti-MBP SMI 99 IgG; lane 8, control antibody IgG. Supernatant fraction was not loaded for the gel used for the actin blot, and the immunoprecipitated supernatant fractions shown in lanes 2 and 3 for the actin blot were from a different gel than the remaining samples, due to overloading and overexposure of the immunoprecipitated supernatant samples on the gel/blot used for the remaining samples. Standards for MBP, tubulin, and actin were not loaded on the gels used for these samples, but can be seen in Figure 2a. The 3 bands for MBP at M_*r*_ values of approximately 16–27 kDa, indicated by arrows, represent the classic 14-, 18.5-, and 21.5-kDa isoforms of MBP. The 14-kDa isoform was not detected with the monoclonal anti-MBP (SMI 99) antibody but was immunoprecipitated by it, since it was detected by E13 antibody in the anti-MBP (SMI 99) immunoprecipitate (lanes 2,5). Lower-exposure blots show the MBP bands in the immunoprecipitates clearly but did not show those in the pellet or supernatant as well. Therefore, a more highly-exposed blot was chosen for this figure.

It has been shown previously, by microscopy of myelin and OLGs extracted with 0.5–1% TX-100 in similar buffers, that structures resembling the myelin radial component and the OLG cytoskeleton were preserved and were in the detergent-insoluble pellet [36,37,53,54]. On the basis of these early studies, therefore, complexes of MBP with cytoskeletal and other proteins are expected here to be in the pellet, in addition to the supernatant in TX-100. Indeed, immunoprecipitation of the TX-100-insoluble pellet with monoclonal anti-MBP (SMI 99) antibody followed by immunoblotting showed specific enrichment of MBP, tubulin, and actin (Figure 1, lane 5) compared to the control immunoprecipitate (Figure 1, lane 6). These results indicate that MBP is associated with tubulin and actin in the TX-100 insoluble fraction. The immunoprecipitation results for the supernatant could not be interpreted due to the similarity of results for the anti-MBP SMI 99 and control immunoprecipitates for both MBP and actin (Figure 1, lanes 2,3). No tubulin was detected in the immunoprecipitates of the supernatant. The lack of specificity of the supernatant immunoprecipitate may be due to the presence of membranous agglomerates not solubilized by TX-100, which may have bound non-specifically to the Dynabeads-Protein G or bound antibodies, even though the samples were pre-cleared first with native Dynabeads-Protein G (see Discussion below).

**Figure 2:**
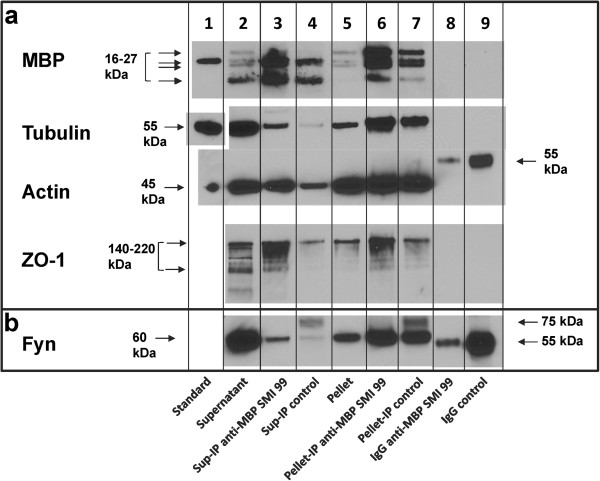
**Second level of stringency – extraction with Triton X-100 and NP40.** MBP, tubulin, actin, ZO-1, and Fyn co-immunoprecipitate from the OLG TX-100 plus NP40-insoluble pellet and supernatant fractions. Primary OLGs were extracted with buffer containing 1% TX-100 plus 1% NP40, and the supernatant and resuspended pellet were immunoprecipitated with mouse monoclonal antibody anti-MBP SMI 99 and a control antibody. Western blots were immunostained with **(a)** rabbit polyclonal anti-MBP (E13), rabbit polyclonal anti-tubulin, mouse monoclonal anti-actin, rat monoclonal anti-ZO-1, and **(b)** mouse monoclonal anti-Fyn. Representative results are shown from 3 experiments for MBP, tubulin, and actin, and from 2 experiments for ZO-1 and Fyn. **(a, b)** lane 1, standard purified protein sample (18.5-kDa isoform for MBP), except for ZO-1 and Fyn (the tubulin sample was run on the same gel as the other lanes, but separated from them by a M_*r*_ marker lane and, therefore, was cut out and shown separately to preserve lane alignment); lane 2, supernatant fraction; lane 3, anti-MBP (SMI 99) immunoprecipitate of supernatant; lane 4, control antibody immunoprecipitate of supernatant; lane 5, pellet; lane 6, anti-MBP (SMI 99) immunoprecipitate of pellet; lane 7, control antibody immunoprecipitate of pellet; lane 8, anti-MBP SMI 99 IgG; lane 9, control antibody IgG. Arrows for the MBP blot indicate the 21.5-, 18.5-, 17-, and 14-kDa isoforms of MBP. Lower-exposure blots showed the MBP bands in the immunoprecipitates clearly, but less well for the pellet or supernatant. Therefore, a more highly-exposed blot was chosen for this figure. The M_*r*_ of ZO-1 is about 220 kDa, but this antibody also detects several unidentified bands down to 110 kDa in rat liver, according to the manufacturer. **(b)** Fyn was detected in the pellet and supernatant at M_*r*_ of about 60 kDa (lanes 2,5).

**Figure 3:**
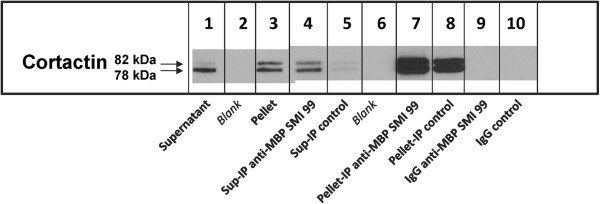
**Second level of stringency – extraction with Triton X-100 and NP40 – cortactin blot.** MBP and cortactin co-immunoprecipitate from the OLG Triton X-100 plus NP40-insoluble pellet and supernatant fractions. Lane 1, supernatant fraction; lane 2, blank; lane 3, pellet; lane 4, anti-MBP (SMI 99) immunoprecipitate of supernatant; lane 5, control antibody immunoprecipitate of supernatant; lane 6, blank; lane 7, anti-MBP (SMI 99) immunoprecipitate of pellet; lane 8, control antibody immunoprecipitate of pellet; lane 9, anti-MBP SMI 99 IgG; lane 10, control antibody IgG. The supernatant and pellet fractions were run on a different gel from the immunoprecipitated samples. The blots were stained with rabbit polyclonal anti-cortactin antibody. Two bands at about 78 kDa and 82 kDa were detected for cortactin. Representative results are shown from 2 experiments.

**Figure 4:**
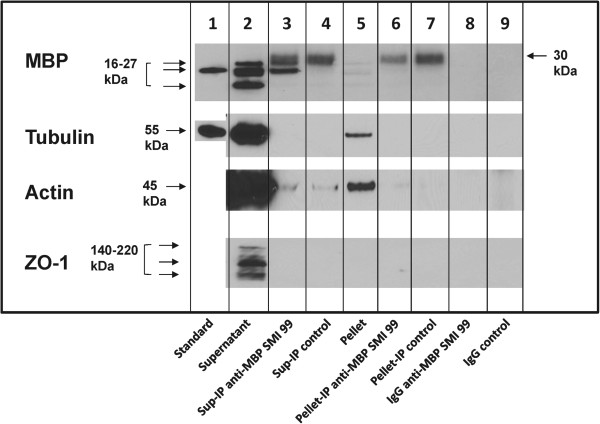
**Third level of stringency – extraction with Triton X-100 and NP40 and DOC.** MBP, tubulin, actin, and ZO-1 do not co-immunoprecipitate in 1% TX-100 plus 1% NP40 plus 1% DOC; addition of DOC disrupts the protein complexes. Primary OLGs were extracted with buffer containing 1% TX-100 plus 1% NP40 plus 1% DOC, the soluble and resuspended insoluble fractions were immunoprecipitated with mouse monoclonal antibody anti-MBP SMI 99 and a control antibody. Western blots were immunostained with rabbit polyclonal anti-MBP (E13), rabbit polyclonal anti-tubulin, mouse monoclonal anti-actin, and rat monoclonal anti-ZO-1 antibodies. Lane 1, standard purified protein sample (18.5-kDa isoform for MBP), except for actin and ZO-1; lane 2, supernatant fraction; lane 3, anti-MBP (SMI 99) immunoprecipitate of supernatant; lane 4, control antibody immunoprecipitate of supernatant; lane 5, pellet; lane 6, anti-MBP (SMI 99) immunoprecipitate of pellet; lane 7, control antibody immunoprecipitate of pellet; lane 8, anti-MBP SMI 99 IgG; lane 9, control antibody IgG.

Despite efforts to prevent the contamination of the immunoprecipitated samples with mouse IgG light chain or heavy chain or their detection on the blots (see Materials and Methods), these bands were sometimes detected by the anti-mouse IgG second antibodies used for some mouse primary antibodies. The following analysis indicates that their presence did not prevent detection or assessment of relative amounts of the OLG proteins of interest. See Additional file 1: Tables S1-S4 for a summary of bands observed in Figures 1, 2, 3, and 4, respectively, and their identification.

Two different anti-MBP antibodies were used for the western blot shown in Figure 1. The western blot with the same mouse anti-MBP SMI 99 antibody used for immunoprecipitation showed two bands for the pellet, supernatant, and the immunoprecipitated samples at M_*r*_ values of approximately 24 and 27 kDa, corresponding to the 18.5-kDa and 21.5-kDa isoforms of MBP. (Highly-charged and intrinsically-disordered proteins such as MBP always run anomalously on SDS-polyacrylamide gels [9,55], and the M_*r*_ values observed were thus as expected.) The light chain from the pure SMI 99 IgG run on the gel was not detected, but that from the control antibody gave a band with M_*r*_ of about 28 kDa (Figure 1, lane 8), slightly above that of the 21.5-kDa MBP isoform. However, in the immunoprecipitated samples detected by mouse anti-MBP SMI 99 and anti-mouse IgG second antibody, another band was also present at M_*r*_ approximately 30 kDa, above that of light chain (Figure 1, lanes 2,3,5,6). This new band could be due to light chain after binding the cross-linking reagent BS^3^, since it was not present in the supernatant or pellet fractions (Figure 1, lanes 1,4) or in the non-cross-linked antibody samples (Figure 1, lanes 7,8).

Use of a polyclonal rabbit anti-MBP antibody (E13) to detect MBP showed 3 MBP bands at about M_*r*_ 16, 24, and 27 kDa, corresponding to 14-, 18.5-, and 21.5-kDa MBP isoforms in all samples [37] (Additional file 1: Table S1). Longer exposure was necessary to detect them in the pellet (not shown). The middle band, identified as 18.5-kDa MBP, migrated similarly to 18.5-kDa MBP isolated from bovine brain (as shown in Figure 2a, for comparison). No IgG bands were detected by the anti-rabbit second antibody used with the rabbit E13 antibody blot for the pure mouse anti-MBP SMI 99 IgG or control IgG applied to the gel (Figure 1, lanes 7, 8), but a faint band with M_*r*_ slightly above that for 21.5-kDa MBP was detected in the immunoprecipitated samples, similar to that detected on the mouse SMI 99 anti-MBP blot. However, the presence of 18.5-kDa and 14-kDa MBP on the anti-MBP E13 blot confirmed the presence of MBP in the immunoprecipitated sample. The anti-MBP SMI antibody (Covance) was raised against a peptide comprising residues 131–136 of 18.5-kDa MBP, which is absent in the 14.0-kDa isoform, and thus this antibody did not detect this smallest isoform.

Bands due to IgG heavy chain, seen in the actin blot in lanes 7 and 8 of Figure 1 where pure mouse IgG was loaded, had an M_*r*_ about 55 kDa, well above that for actin, and were not detected in the immunoprecipitated samples for actin and tubulin in any case. An anti-rabbit IgG was used for the rabbit anti-tubulin blot, and did not detect the mouse heavy chain used for immunoprecipitation.

#### ***Extraction and immunoprecipitation with buffers containing 1% TX-100 plus 1% NP-40***

The complexes that are immunoprecipitated from the pellet of a TX-100 lysate could be membranous, *e.g.* lipid-ordered membrane domains, which have been found to be complexed to cytoskeletal proteins in TX-100 extracts of myelin and have low buoyant density on a sucrose density gradient [56]. Therefore, 1% NP-40 was then added to the TX-100-containing buffer, for lysis and immunoprecipitation, and the polyclonal rabbit anti-MBP E13 antibody was used to analyze for MBP (Figure 2a and Additional file 1: Table S2). In this blot, the 17-kDa isoform of MBP can also be detected due to higher resolution on the gel (Figure 2a, lanes 2,5). Several SH3-domain proteins that have been shown previously to bind to MBP *in vitro*, or be co-localized with it in cells, namely Fyn, ZO-1, and cortactin, were also analyzed [20,32,39]. In 1% TX-100 plus 1% NP-40, the anti-MBP SMI 99 immunoprecipitates of both the supernatant and the detergent-insoluble pellet were enriched in MBP, actin, tubulin, Fyn, ZO-1, and cortactin compared to the control immunoprecipitate (Figure 2a, b, lanes 3,6 compared to lanes 4,7; Figure 3, lanes 4,7 compared to lanes 5,8). The heavy chain of the mouse antibodies (55 kDa) was detected by the anti-mouse IgG used for the mouse anti-Fyn blot, but it had a sufficiently lower M_*r*_ than Fyn (60 kDa) and thus did not interfere (Figure 2b, lanes 8,9). A band with M_*r*_ of about 75 kDa, higher than that of Fyn, in the control antibody-immunoprecipitated fractions (Figure 2b, lanes 4,7) that was not present in the pure IgG samples or in the supernatant and pellet fractions, is most likely due to the cross-linked light chain plus heavy chain. No IgG bands were detected for pure IgG samples in the ZO-1 (Figure 2b, lanes 8,9) or cortactin (Figure 3, lanes 9,10) blots or in the immunoprecipitated samples (Additional file 1: Tables S2 and S3).

Thus, protein complexes containing MBP, actin, tubulin, Fyn, ZO-1, and cortactin, were specifically precipitated by anti-MBP antibody from both the pellet and the supernatant fractions in TX-100 plus NP-40. The observation of greater binding of other proteins to anti-MBP-bound beads, compared to control antibody-bound beads, was reproducible in replicate experiments. The greater precipitation of MBP, actin, and tubulin from the pellet in TX-100 plus NP40 by anti-MBP, compared to that by the control antibody, agrees with results obtained for the pellet in TX-100 alone.The difference between anti-MBP-precipitated and control antibody-precipitated protein was even greater for MBP, actin, and tubulin in the supernatant fraction than the pellet fraction in TX-100 plus NP40, in contrast to results for TX-100 alone, indicating that the MBP/actin/tubulin-containing protein complexes in the supernatant were better solubilized by the combined detergents, and thus bound less non-specifically to control antibody or beads. More of the MBP-containing complexes may also have transferred from the pellet to the supernatant in the presence of both detergents, probably due to release from membranes. Since the MBP complexes are better solubilized by the combined detergents, TX-100 and NP-40, this result supports the occurrence of direct protein-protein interactions rather than binding to non-specific membranous agglomerates, although it is possible that other proteins are also required to mediate the interactions. The proteins were not all enriched to the same extent in the immunoprecipitated material. Both MBP and ZO-1 were enriched in the supernatant immunoprecipitate relative to the other proteins. In Figures 2 and 3, MBP, tubulin, ZO-1, and Fyn are seen to be enriched in the pellet immunoprecipitate relative to actin and cortactin. This observation suggests that the MBP complexes are heterogeneous, with some MBP molecules interacting with only some of the detected proteins, and other MBP molecules interacting with others.

#### ***Extraction and immunoprecipitation with TX-100 plus NP-40 plus DOC***

However, when 1% DOC was also added to the buffer containing TX-100 and NP-40, and used for cell lysis and immunoprecipitation, most of the MBP, actin, tubulin and ZO-1 were extracted into the supernatant (Figure 4, lane 2 *versus* lane 5), and little or none of the other proteins besides MBP was detected in the immunoprecipitated supernatant or pellet samples (Figure 4, and Additional file 1: Table S4). A trace of actin was detected in the anti-MBP immunoprecipitate of the supernatant, but was also present in the control immunoprecipitate (Figure 4, lanes 3,4). Thus, we conclude that DOC disrupted most of the protein complexes containing MBP, which had remained intact in the presence of the milder detergents TX-100 and NP-40.

### Co-localization of MBP and actin in N19-OLGs after recovery from cytochalasin

The conditionally-immortalized N19-OLG cell line closely resembles an immature OLG, before MBP begins to be synthesised in large quantities [57,58]. As such, it has represented an ideal model system to study the trafficking and interactions of classic 18.5-kDa and 21.5-kDa MBP isoforms after transfection [32,39,50,59,60]. The plasmid encoding all GFP/RFP-fusion MBP variants had a 21-nucleotide 3′UTR which was found to be essential to ensure that the mRNA encoding the 18.5-kDa isoform was trafficked to the cell periphery, after which the resultant protein was incorporated into the plasma membrane, in contrast to proteins lacking the signal, and in contrast to control vectors encoding either GFP or RFP alone [32,50]. In these studies, the membrane-association of classic 18.5-kDa MBP variants was found to be essential for several phenotypic effects in early developmental N19-OLGs that did not occur with controls expressing GFP or RFP alone, specifically:

(i) inhibiting calcium influx by voltage-operated calcium channels (VOCCs), induced by membrane depolarization caused by high extracellular [K^+^] [50];

(ii) interacting with the SH3-domain of Fyn, with physiological effects such as membrane process extension [32];

(iii) co-localization with cytoskeletal proteins in N19-cells co-transfected with fluorescently-tagged MBP, actin, and tubulin [39].

In this latter study, we observed additionally that this classic 18.5-kDa MBP isoform co-localized with the SH3-domain-containing proteins cortactin and ZO-1, when stimulated with PMA. The focus in these experiments was on membrane ruffles that were induced by exogenous PMA, and which contained membrane-associated MBP variants fused with RFP or GFP. When RFP or GFP were expressed alone, they did not traffic to the cell periphery.

We have extended the investigations in reference [39] as follows. Here, N19-OLGs transfected with RFP-MBP and GFP-β-actin were exposed to 2 μM CytD for 2 hours to depolymerize the cytoskeleton [51,52], and then allowed to recover in its absence, in order to examine the redistribution of MBP and actin during repolymerization of the actin cytoskeleton. Cytochalasin D is a fungal metabolite that causes reversible depolymerization of actin filaments, and is widely used to study the role of actin in biological processes [51,52]. Its depolymerizing effect on actin filaments in cells is reversed on removing it from the medium in which the cells are incubated.

**Figure 5:**
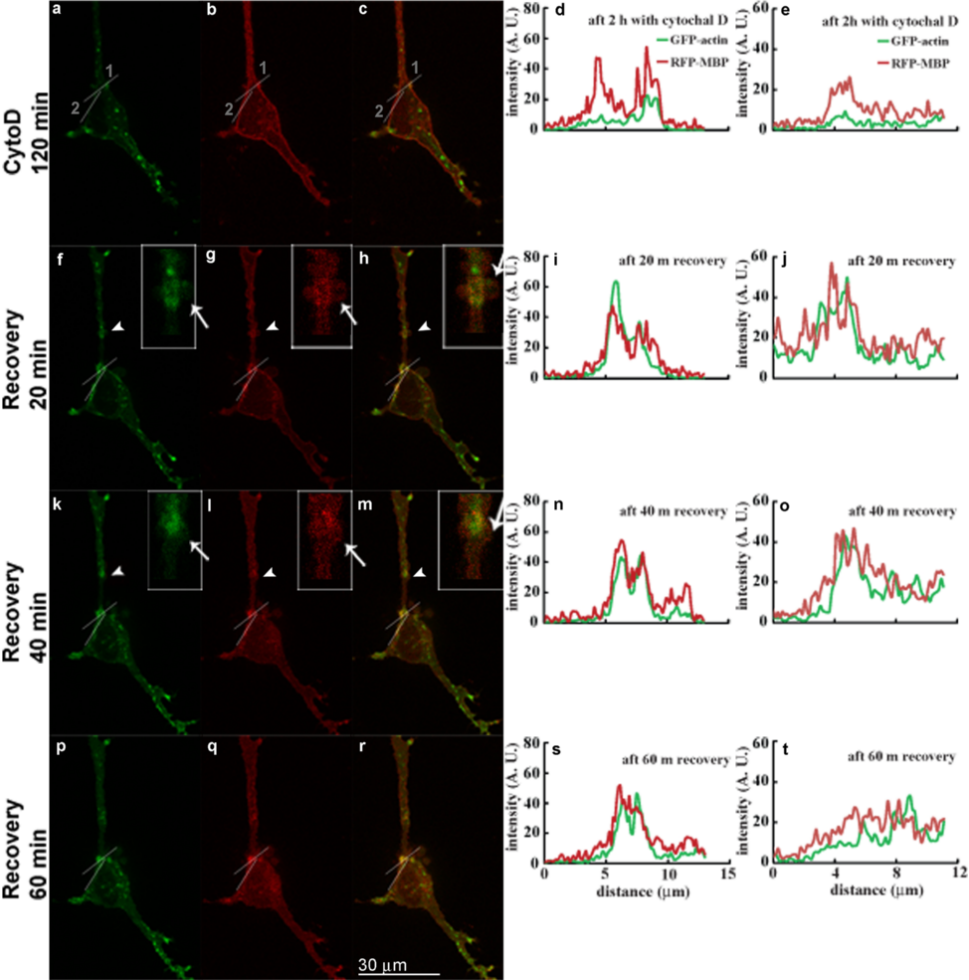
**Live cell images of N19-OLGs treated with CytD for 2 hours, and then allowed to recover in its absence.** The N19-OLGs after incubation with CytD for 2 hours **(a-e)**, after 20 min recovery **(f-j)**, after 40 min recovery **(k-o)**, and after 60 min recovery **(p-t)**. Panels **a**, **f**, **k**, and **p** show GFP-actin signal; panels **b**, **g**, **l**, and **q** show RFP-MBP signal; panels **c**, **h**, **m**, and **r** show the merge of GFP-actin and MBP-RFP signals; panels **d**, **i**, **n**, and **s** correspond to intensity plots along line 1, and panels **e**, **j**, **o**, and **t** represent intensity plots along line 2 for GFP-actin (green) and RFP-MBP (red). The intensity plots are in arbitrary units and do not quantitatively reflect the MBP and actin enrichment at the sites analyzed, but show changes in relative intensity of MBP and actin, their location and their degree of co-localization with time. The arrowheads in white indicate regions where ruffles were observed after removing CytD. The regions with ruffles are shown at larger size in the inset in panels **f**, **g**, **h**, **k**, **l**, and **m**, and the ruffles are indicated in the insets by arrows. Scale bar represents 30 μm.

**Figure 6:**
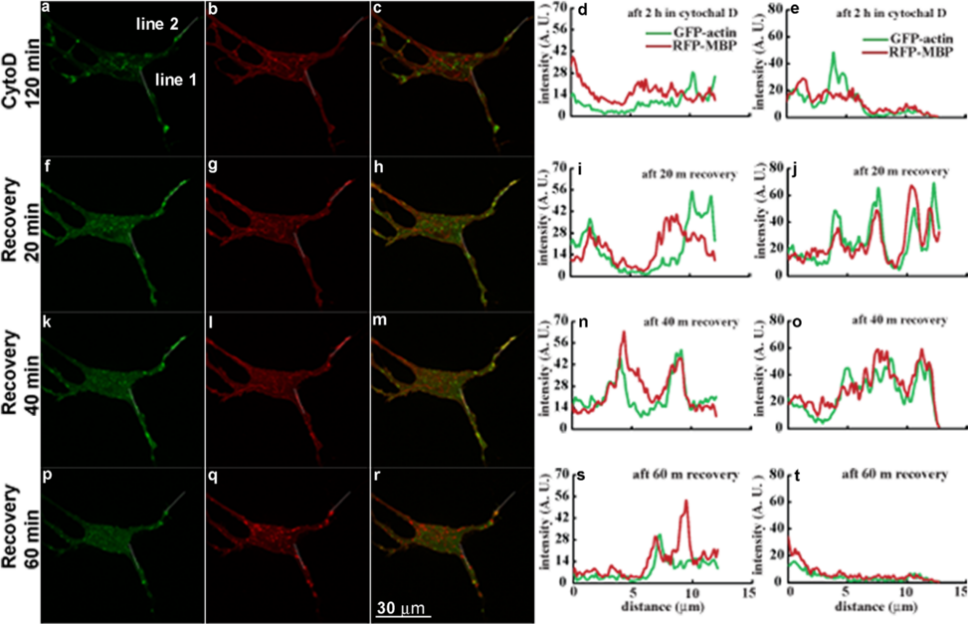
**Live-cell images of another N19-OLG treated with CytD for 2 hours (a-e), after 20 min recovery (f-j), after 40 min recovery (k-o), and after 60 min recovery (p-t).** Panels **a**, **f**, **k**, and **p** show GFP-actin signal; panels **b**, **g**, **l**, and **q** show RFP-MBP signal; panels **c**, **h**, **m**, and **r** show the merge of GFP-actin and MBP-RFP signals; panels **d**, **i**, **n**, and **s** correspond to intensity plots along line 1, and panels **e**, **j**, **o**, and **t** represent intensity plots along line 2 for GFP-actin (green) and RFP-MBP (red). The intensity plots are in arbitrary units and do not quantitatively reflect the MBP and actin enrichment at the sites analyzed, but show changes in relative intensity of MBP and actin, their location and their degree of co-localization with time. The arrowheads in white indicate regions where ruffles were observed after removing CytD. Scale bar represents 30 μm.

Figure 5 and Figure 6 show live-cell fluorescence images of two different N19-OLGs co-transfected with GFP-β-actin and RFP-MBP after 2 hours exposure to CytD (panels a-c); 20 min after its removal from the cell medium (panels f-h); 40 min after its removal (panels k-m); and 60 min after its removal (panels p-r). Treatment with CytD for 2 hours caused depolymerization of actin filaments and formation of aggregates of GFP-β-actin both in the cell body and in cell processes (Figure 5a, Figure 6a). The RFP-MBP, on the other hand, is mostly localized at the plasma membrane along the edges of the cell (Figure 5b, Figure 6b). Here, we followed the protocol of Peyrollier et al. [52] who treated their cells for 2 hours, and who confirmed then using rhodamine-phalloidin staining that actin depolymerization had, indeed, occurred. In preparatory control experiments, we established that 2 hours was optimal for effecting depolymerization of actin filaments and for appearance of clumps of monomeric actin in this N19-cell system (not shown) – these experiments were performed on fixed cells, both transfected with RFP-MBP and untransfected, and stained with rhodamine-phalloidin. Other precedents in the literature report 2–4 hours of CytD exposure of primary OLGs to be most efficacious in depolymerizing actin microfilaments [34,35]. Here, the changes in the GFP signal confirmed further the depolymerization upon exposure to the drug.Removal of CytD from the N19-cell medium results in a more diffuse distribution of actin filaments at the plasma membrane. Actin filaments are also distributed in membrane ruffles formed 20–40 min after removing CytD from the cell medium (indicated by white arrowheads in Figure 5f, k). The ruffled region is shown enlarged in insets in Figure 5h, k-m, and the ruffles are indicated by white arrows in the insets. Since the actin fluorescence intensity is so low in the presence of CytD, the most reasonable explanation for the increase of actin-GFP intensity after drug removal is simply the polymerization of actin filaments, as confirmed by rhodamine-phalloidin staining of fixed cells.Membrane ruffles are regions of transient actin assembly and dis-assembly and their occurrence during the repolymerization of actin is not unusual. However, in this case, they were not observed in all the cells studied. Close inspection of the ruffles indicates rapid co-localization of MBP with actin in these regions (Figure 5, panels f-h, k-m). The MBP and actin intensity also both increased at the same time after repolymerization of actin filaments at certain sites at the plasma membrane. Line-intensity profiles measured along two different lines plotted across the plasma membrane at these sites show increased co-localization of MBP and actin at these sites in the first 20–40 min after recovery (Figure 5i, j, n, o), compared to the pattern after incubation with CytD (Figure 5d, e). Since the intensity is plotted in arbitrary units (AU), the increase in absolute intensity is not always obvious in the line-intensity profiles, although an increase is seen in Figures 5j and 6i, j, n, o, s, with a decrease in Figure 6t at 60 min. Both MBP and actin distribution simultaneously change at one site (Figure 5i, n in comparison to Figure 5d) and a new peak of co-localized MBP and actin intensity appears at 40 min (Figure 5n), whereas MBP and actin increase in intensity at the other site at 20–40 min (Figure 5e, j, o). At 60 min after recovery from CytD treatment, however, there is a drop in intensity of MBP and actin at one site (Figure 5t) but not the other site (Figure 5s), and membrane ruffles are diminished.In this work, ten different cells were examined in several different experiments. The examples shown in Figure 5 and Figure 6 for two different cells are representative and in agreement with each other. In Figure 6, changes in both MBP and actin distribution occurred at both sites analyzed in this second cell, and the MBP and actin increase in intensity and become co-localized at the plasma membrane by 20–40 min after CytD removal. By 60 min, the intensity of both MBP and actin and the degree of co-localization at these sites had decreased, indicating dynamic changes in the distribution of both proteins and of the cytoskeleton occurring during this time period. Thus, there is a marked change in distribution and co-localization of actin and MBP in these cells by 20 min after removal of CytD when the actin cytoskeleton undergoes repolymerization. It is possible in Figure 5 and Figure 6 that the MBP and actin may be newly-synthesized, but if so, MBP is enriched at the sites of actin repolymerization and/or synthesis, suggesting that the two proteins interact.

## Discussion

We have shown here that classic MBP isoforms are specifically co-immunoprecipitated from primary OLGs in complexes with actin, tubulin, and several SH3-domain proteins (Fyn, ZO-1, and cortactin). In 1% TX-100, the MBP/actin/tubulin complexes were present in the pellet obtained by centrifugation at 14,000 g, but were non-specifically bound by the control antibody beads from the supernatant. They were more efficiently solubilized in the supernatant by both 1% TX-100 and 1% NP-40, allowing their specific immunoprecipitation from the supernatant in addition to the pellet. Addition of 1% DOC to this detergent mixture disrupted these protein complexes. Although NP-40 is similar to TX-100, it is less hydrophilic than TX-100 (Sigma product data sheet) and may solubilize lipid better and/or release protein complexes from membranous domains. In contrast, DOC is a harsher, denaturing detergent which can disrupt protein-protein interactions. Different degrees of enrichment of different proteins in the immunoprecipitated material indicate that the MBP complexes are heterogeneous.

The 18.5-kDa MBP isoform is a small, highly-basic protein that interacts electrostatically with negatively-charged proteins such as actin and tubulin, and with the SH3-domain proteins also through its single SH3-ligand domain at its proline-rich region comprising amino acids T92-S99 (murine 18.5-kDa sequence numbering) [9,10]*.* Although these proteins all bind directly to MBP *in vitro* (reviewed in [27]), it is unlikely that such a small protein can interact with all of these other proteins at once *in vivo*[61]. For instance, we have shown *in vitro* that it can bind actin filaments and microtubules to each other [26], but its simultaneous binding to more than one of the other proteins has not been investigated. Most likely here, some molecules of MBP in the immunoprecipitated material are bound to some of the proteins that are detected, and other molecules are bound to the other proteins that are detected.

These proteins may have been bound indirectly to MBP through other proteins or through lipids, even when in the supernatant. This may explain why significant amounts of these proteins were also in the control immunoprecipitate. Since MBP binds tightly to negatively-charged lipids and can bind actin filaments, microtubules, and the SH3-domain protein Fyn to a lipid bilayer [24-26], it is quite likely that these immunoprecipitated complexes are large and bound to lipid domains. Such large complexes would be quite likely to bind non-specifically to control IgG or beads in addition to specific binding to anti-MBP. The fact that the amounts of all proteins precipitated were reproducibly greater in the anti-MBP immunoprecipitate, than in the control immunoprecipitate, indicates that a significant proportion of them were complexed with MBP.

The immunoprecipitation data here are supported further by additional live-cell fluorescence microscopy experiments performed on cultured, transfected N19-OLG cells, extending an earlier and more comprehensive study [39]. We had shown then by fluorescence microscopy that 18.5-kDa MBP transfected into N19-OLGs co-localized with actin, tubulin, and the SH3-domain-containing proteins cortactin and ZO-1, in membrane ruffles when stimulated with PMA [39]. Moreover, it was co-localized with Fyn in the cell body and process tips. Here, we have also shown that actin filaments formed soon after removal of CytD are co-localized with MBP at sites at the plasma membrane and in new membrane ruffles of N19-OLGs. Both MBP and actin intensity are increased at these sites, indicating that both proteins redistribute to new sites where they are co-localized when actin repolymerization occurs. This observation supports our conclusions from previous studies of transfected N19-OLGs showing that MBP and actin associate in newly-formed membrane ruffles after stimulation with PMA, and at membrane domains resembling focal adhesion contacts formed after stimulation with IGF-1 [39].

It is rare for co-immunoprecipitation studies alone to prove simply and unequivocally that specific protein-protein interactions actually do occur *in vivo *[62-64]. The immunoprecipitation and microscopy data presented here together thus support the thesis that these associations also occur in living cells. It is acknowledged that the resolution of light microscopy is on the order of 0.5 μm and a technique such as FRET (fluorescence resonance energy transfer) might be able in future studies to demonstrate direct binding of two proteins to each other in the complexes *in vivo*. Nonetheless, the facts that they bind *in vitro*, are co-immunoprecipitated from primary OLGs, and, in the case of actin and MBP, redistribute to and are co-localized to the same regions of living cells at similar times after repolymerization (and/or new synthesis) of actin filaments support the conclusion that they are likely present together with MBP in the same membrane domains, where MBP may be able to influence their behavior and activities, as we have previously reviewed [27]. Moreover, the extensive myelin fractionation literature is also wholly consistent with our immunoprecipitation results here on primary OLGs. Classic MBP isoforms have been found to be enriched in TX-100 insoluble, low density membrane domains from myelin that contain glycosphingolipids, actin, tubulin, Fyn, and other proteins [56]. Similar enrichments of MBP isoforms with Fyn have been shown in CHAPS-resistant myelin microdomains [65,66]. These associations can be biologically significant in two ways as described next.

First, Fyn is a member of the Src family of tyrosine kinases with important roles in OLG differentiation and myelination (reviewed in reference [67]). Cortactin is an actin-binding protein that plays a role in regulation of actin dynamics in cell lamellipodia and ruffles [68]. The scaffold protein ZO-1 is associated with numerous signaling proteins, tight and gap junctions, and the actin cytoskeleton [69]. It may be associated with gap junctions in OLGs [70,71], and in the radial component of myelin, which contains other tight junction proteins [72]. The radial component of myelin is TX-100-insoluble along with MBP (especially the 17-kDa and 21.5-kDa isoforms), actin, tubulin, CNP, and glycosphingolipids [53,54], further suggesting an association of MBP with tight junction proteins. Interactions of classic MBP isoforms with the cytoskeleton, ZO-1, and other SH3-domain proteins such as Fyn may allow it to participate in regulation of junctional activity [69].

Second, many myelination events, such as OLG process extension, membrane sheet formation, and ensheathment of the axon depend on dynamic changes in the cytoskeleton [73,74]. Association of actin and tubulin with MBP during dynamic changes in the cytoskeleton, as demonstrated here after actin repolymerization, points to a functional interaction between the two proteins, and implicates diverse structural and networking roles for MBP during myelin formation and turnover [27]. Even adult myelin is a highly-dynamic structure [75], and it can be expected that an essential protein such as classic MBP would participate in local cytoskeletal remodeling [73,76], making it another notable example of intrinsically-disordered accessory proteins responsible for the dynamic regulation and adaptation of cytoskeletal systems [77-81].

## Conclusions

The co-immunoprecipitation of MBP with actin, tubulin, Fyn-kinase, ZO-1 and cortactin from primary OLGs, and the co-localization results from N19-OLGs showing that both actin and MBP re-associate on actin polymerization and that both proteins rapidly redistribute to new sites of actin polymerization, provide more direct evidence for association of MBP with these proteins in primary OLGs and in live cells, and reinforce earlier conclusions that these proteins associate in cells as found *in vitro* using purified proteins.

## Abbreviations

BS^3^: Bis(sulfosuccinimidyl) suberate; CNP: 2′,3′-cyclic nucleotide 3′-phosphodiesterase; CNS: Central nervous system; CytD: Cytochalasin D; DMEM: Dulbecco’s Modified Eagle Medium; DOC: Sodium deoxycholate; FBS: Foetal bovine serum; Fyn: Member of Src family of tyrosine protein kinases; GFP: Green fluorescent protein; HEPES: *N*-(2-hydroxyethyl) piperazine-*N’*-2-ethanesulfonic acid; HRP: Horseradish peroxidase; IGF-1: Insulin-like growth factor-1; IgG: Immunoglobulin; MAPs: Microtubule-associated proteins; MBP: Myelin basic protein; MES: 2-(*N*-morpholino)ethanesulfonic acid; MOPS: 3-(*N*-morpholino)propanesulfonic acid; NP-40: Nonidet P-40; OLG: Oligodendrocyte; PMA: Phorbol-12-myristate-13-acetate; RFP: Red fluorescent protein; SH3: *Src* homology 3; TBS: Tris-buffered saline; TX-100: Triton X-100; UTR: Untranslated region; ZO-1: Zona occludens 1.

## Competing interests

The authors declare that they have no competing interests.

## Authors’ contributions

LH performed the co-localization experiments in N19-OLGs, GR performed the immunoprecipitation and western blotting, YL cultured the primary OLGs and prepared cell lysates, GSTS provided the plasmids coding for RFP-tagged MBP and GFP-tagged actin, and JMB and GH conceived of the study, designed it, and drafted the manuscript. All authors read and approved the final manuscript.

## Supplementary Material

Additional file 1 The supplementary files available in the online version of this paper comprise Figure S1 (flowchart of experimental procedure) and Tables S1-S4 (interpretations of Western blots in Figures 1–4, respectively). Click here for file

## References

[B1] Readhead C Takasashi N Shine HD Saavedra R Sidman R Hood L Role of myelin basic protein in the formation of central nervous system myelin Ann N Y Acad Sci 1990 605 280 285 1702601 10.1111/j.1749-6632.1990.tb42401.x

[B2] Trapp BD Kidd GJ Lazzarini RA, Griffin JW, Lassman H, Nave K-A, Miller RH, Trapp BD Structure of the Myelinated Axon Myelin Biology and Disorders. Volume 1 2004 San Diego: Elsevier Academic Press 3 27

[B3] Aggarwal S Yurlova L Snaidero N Reetz C Frey S Zimmermann J Pähler G Janshoff A Friedrichs J Muller DJ Goebel C Simons M A size barrier limits protein diffusion at the cell surface to generate lipid-rich myelin-membrane sheets Dev Cell 2011 21 445 456 21885353 10.1016/j.devcel.2011.08.001

[B4] Simons M Snaidero N Aggarwal S Cell polarity in myelinating glia: from membrane flow to diffusion barriers Biochim Biophys Acta 1821 2012 1146 1153 10.1016/j.bbalip.2012.01.011 22314181

[B5] Aggarwal S Snaidero N Pahler G Frey S Sanchez P Zweckstetter M Janshoff A Schneider A Weil MT Schaap IA Gorlich D Simons M Myelin membrane assembly is driven by a phase transition of myelin basic proteins into a cohesive protein meshwork PLoS Biol 2013 11 e1001577 23762018 10.1371/journal.pbio.1001577 PMC3676292

[B6] Bakhti M Aggarwal S Simons M Myelin architecture: zippering membranes tightly together Cell Mol Life Sci 2013 71 1265 1277 24165921 10.1007/s00018-013-1492-0 PMC11113231

[B7] Harauz G Libich DS Polverini E Vassall KA Dunn BM The Classic Protein of Myelin - Conserved Structural Motifs and the Dynamic Molecular Barcode Involved in Membrane Adhesion, Protein-Protein Interactions, and Pathogenesis in Multiple Sclerosis Advances in Protein and Peptide Science 2013 1 Bentham Science Publishers 1 53 e-book; http://benthamscience.com/ebooks/9781608054879/index.htm

[B8] Sedzik J Kirschner DA Is myelin basic protein crystallizable? Neurochem Res 1992 17 157 166 1371603 10.1007/BF00966794

[B9] Harauz G Ishiyama N Hill CMD Bates IR Libich DS Farès C Myelin basic protein - diverse conformational states of an intrinsically unstructured protein and its roles in myelin assembly and multiple sclerosis Micron 2004 35 503 542 15219899 10.1016/j.micron.2004.04.005

[B10] Harauz G Libich DS The classic basic protein of myelin - conserved structural motifs and the dynamic molecular barcode involved in membrane adhesion and protein-protein interactions Curr Protein Pept Sci 2009 10 196 215 19519451 10.2174/138920309788452218

[B11] Harauz G Ladizhansky V Boggs JM Structural polymorphism and multifunctionality of myelin basic protein Biochemistry 2009 48 8094 8104 19642704 10.1021/bi901005f

[B12] Libich DS Ahmed MA Zhong L Bamm VV Ladizhansky V Harauz G Fuzzy complexes of myelin basic protein: NMR spectroscopic investigations of a polymorphic organizational linker of the central nervous system Biochem Cell Biol 2010 88 143 155 Special issue on Protein Folding: Principles and Diseases 20453917 10.1139/o09-123 PMC3517781

[B13] Polverini E Coll EP Tieleman DP Harauz G Conformational choreography of a molecular switch region in myelin basic protein - Molecular dynamics shows induced folding and secondary structure type conversion upon threonyl phosphorylation in both aqueous and membrane-associated environments Biochim Biophys Acta 1808 2011 674 683 10.1016/j.bbamem.2010.11.030 21130728

[B14] Vassall KA Bessonov K De Avila M Polverini E Harauz G The effects of threonine phosphorylation on the stability and dynamics of the central molecular switch region of 18.5-kDa myelin basic protein PLoS One 2013 8 e68175-1-19 23861868 10.1371/journal.pone.0068175 PMC3702573

[B15] Ahmed MAM De Avila M Polverini E Bessonov K Bamm VV Harauz G Solution NMR structure and molecular dynamics simulations of murine 18.5-kDa myelin basic protein segment (S72-S107) in association with dodecylphosphocholine micelles Biochemistry 2012 51 7475 7487 22947219 10.1021/bi300998x

[B16] Boggs JM Myelin basic protein: a multifunctional protein Cell Mol Life Sci 2006 63 1945 1961 16794783 10.1007/s00018-006-6094-7 PMC11136439

[B17] Boggs JM Rangaraj G Gao W Heng YM Effect of phosphorylation of myelin basic protein by MAPK on its interactions with actin and actin binding to a lipid membrane *in vitro* Biochemistry 2006 45 391 401 16401070 10.1021/bi0519194

[B18] Boggs JM Myelin Basic Protein 2008 Hauppauge, NY: Nova

[B19] Libich DS Harauz G Backbone dynamics of the 18.5-kDa isoform of myelin basic protein reveals transient alpha-helices and a calmodulin-binding site Biophys J 2008 94 4847 4866 18326633 10.1529/biophysj.107.125823 PMC2397351

[B20] Polverini E Rangaraj G Libich DS Boggs JM Harauz G Binding of the proline-rich segment of myelin basic protein to SH3-domains - Spectroscopic, microarray, and modelling studies of ligand conformation and effects of post-translational modifications Biochemistry 2008 47 267 282 18067320 10.1021/bi701336n

[B21] Wang C Neugebauer U Burck J Myllykoski M Baumgartel P Popp J Kursula P Charge isomers of myelin basic protein: structure and interactions with membranes, nucleotide analogues, and calmodulin PLoS One 2011 6 e19915 21647440 10.1371/journal.pone.0019915 PMC3102069

[B22] Majava V Petoukhov MV Hayashi N Pirila P Svergun DI Kursula P Interaction between the C-terminal region of human myelin basic protein and calmodulin: analysis of complex formation and solution structure BMC Struct Biol 2008 8 10 18284662 10.1186/1472-6807-8-10 PMC2288786

[B23] Nagulapalli M Parigi G Yuan J Gsponer J Deraos G Bamm VV Harauz G Matsoukas J de Planque MR Gerothanassis IP Babu MM Luchinat C Tzakos AG Recognition pliability is coupled to structural heterogeneity: a calmodulin intrinsically disordered binding region complex Structure 2012 20 522 533 22405011 10.1016/j.str.2012.01.021

[B24] Boggs JM Rangaraj G Interaction of lipid-bound myelin basic protein with actin filaments and calmodulin Biochemistry 2000 39 7799 7806 10869185 10.1021/bi0002129

[B25] Homchaudhuri L Polverini E Gao W Harauz G Boggs JM Influence of membrane surface charge and post-translational modifications to myelin basic protein on its ability to tether the Fyn-SH3 domain to a membrane *in vitro* Biochemistry 2009 48 2385 2393 19178193 10.1021/bi8022587

[B26] Boggs JM Rangaraj G Heng YM Liu Y Harauz G Myelin basic protein binds microtubules to a membrane surface and to actin filaments *in vitro*: Effect of phosphorylation and deimination Biochim Biophys Acta 1808 2011 761 773 10.1016/j.bbamem.2010.12.016 21185260

[B27] Harauz G Boggs JM Myelin management by the 18.5-kDa and 21.5-kDa classic myelin basic protein isoforms J Neurochem 2013 125 334 361 23398367 10.1111/jnc.12195 PMC3700880

[B28] Homchaudhuri L De Avila M Nilsson SB Bessonov K Smith GST Bamm VV Musse AA Harauz G Boggs JM Secondary structure and solvent accessibility of a calmodulin-binding C-terminal segment of membrane-associated myelin basic protein Biochemistry 2010 49 8955 8966 20831157 10.1021/bi100988p

[B29] Libich DS Hill CMD Bates IR Hallett FR Armstrong S Siemiarczuk A Harauz G Interaction of the 18.5-kDa isoform of myelin basic protein with Ca2 + -calmodulin: effects of deimination assessed by intrinsic Trp fluorescence spectroscopy, dynamic light scattering, and circular dichroism Protein Sci 2003 12 1507 1521 12824496 10.1110/ps.0303603 PMC2323942

[B30] Musse AA Boggs JM Harauz G Deimination of membrane-bound myelin basic protein in multiple sclerosis exposes an immunodominant epitope Proc Natl Acad Sci U S A 2006 103 4422 4427 16537438 10.1073/pnas.0509158103 PMC1450187

[B31] Harauz G Musse AA A tale of two citrullines - structural and functional aspects of myelin basic protein deimination in health and disease Neurochem Res 2007 32 137 158 16900293 10.1007/s11064-006-9108-9

[B32] Smith GST De Avila M Paez PM Spreuer V Wills MKB Jones N Boggs JM Harauz G Proline substitutions and threonine pseudophosphorylation of the SH3 ligand of 18.5-kDa myelin basic protein decrease its affinity for the Fyn-SH3 domain and alter process development and protein localization in oligodendrocytes J Neurosci Res 2012 90 28 47 21887699 10.1002/jnr.22733 PMC3527418

[B33] Boggs JM Rangaraj G Dicko A Effect of phosphorylation of phosphatidylinositol on myelin basic protein-mediated binding of actin filaments to lipid bilayers *in vitro* Biochim Biophys Acta 1818 2012 2217 2227 10.1016/j.bbamem.2012.04.006 22538354

[B34] Dyer CA Benjamins JA Organization of oligodendroglial membrane sheets. I: association of myelin basic protein and 2′,3′-cyclic nucleotide 3′-phosphohydrolase with cytoskeleton J Neurosci Res 1989 24 201 211 2479763 10.1002/jnr.490240211

[B35] Dyer CA Benjamins JA Organization of oligodendroglial membrane sheets: II. Galactocerebroside:antibody interactions signal changes in cytoskeleton and myelin basic protein J Neurosci Res 1989 24 212 221 2479764 10.1002/jnr.490240212

[B36] Gillespie CS Wilson R Davidson A Brophy PJ Characterization of a cytoskeletal matrix associated with myelin from rat brain Biochem J 1989 260 689 696 2764898 10.1042/bj2600689 PMC1138732

[B37] Wilson R Brophy PJ Role for the oligodendrocyte cytoskeleton in myelination J Neurosci Res 1989 22 439 448 2474666 10.1002/jnr.490220409

[B38] Dyer CA Philibotte TM Wolf MK Billings-Gagliardi S Myelin basic protein mediates extracellular signals that regulate microtubule stability in oligodendrocyte membrane sheets J Neurosci Res 1994 39 97 107 7528819 10.1002/jnr.490390112

[B39] Smith GST Homchaudhuri L Boggs JM Harauz G Classic 18.5- and 21.5-kDa myelin basic protein isoforms associate with cytoskeletal and SH3-domain proteins in the immortalized N19-oligodendroglial cell line stimulated by phorbol ester and IGF-1 Neurochem Res 2012 37 1277 1295 22249765 10.1007/s11064-011-0700-2 PMC3527419

[B40] Dyer CA Philibotte TM Billings-Gagliardi S Wolf MK Cytoskeleton in myelin-basic-protein-deficient *shiverer* oligodendrocytes Dev Neurosci 1995 17 53 62 7542583 10.1159/000111273

[B41] Galiano MR Andrieux A Deloulme JC Bosc C Schweitzer A Job D Hallak ME Myelin basic protein functions as a microtubule stabilizing protein in differentiated oligodendrocytes J Neurosci Res 2006 84 534 541 16773649 10.1002/jnr.20960

[B42] Galiano MR Lopez Sambrooks CH Lak ME Boggs JM Insights into the Interaction of Myelin Basic Protein With Microtubules Myelin Basic Protein 2008 New York: Nova 129 147

[B43] Galiano MR Hallak ME Phosphorylation of MBP Increased its Interaction With Microtubules in Oligodendrocytes 2008 Washington, DC: Neuroscience Meeting Proceedings

[B44] Taketomi M Kinoshita N Kimura K Kitada M Noda T Asou H Nakamura T Ide C Nogo-A expression in mature oligodendrocytes of rat spinal cord in association with specific molecules Neurosci Lett 2002 332 37 40 12377379 10.1016/s0304-3940(02)00910-2

[B45] Kozielski F Riaz T Debonis S Koehler CJ Kroening M Panse I Strozynski M Donaldson IM Thiede B Proteome analysis of microtubule-associated proteins and their interacting partners from mammalian brain Amino Acids 2011 41 363 385 20567863 10.1007/s00726-010-0649-5

[B46] Kang EY Ponzio M Gupta PP Liu F Butensky A Gutstein DE Identification of binding partners for the cytoplasmic loop of connexin43: a novel interaction with beta-tubulin Cell Commun Adhes 2009 15 397 406 19274588 10.1080/15419060902783833 PMC2889002

[B47] Boggs JM Samji N Moscarello MA Hashim GA Day ED Immune lysis of reconstituted myelin basic protein-lipid vesicles and myelin vesicles J Immunol 1983 130 1687 1694 6187818

[B48] Arnold T Linke D Phase separation in the isolation and purification of membrane proteins Biotechniques 2007 43 427 430 432, 434 18019333 10.2144/000112566

[B49] Boggs JM Wang H Co-clustering of galactosylceramide and membrane proteins in oligodendrocyte membranes on interaction with polyvalent carbohydrate and prevention by an intact cytoskeleton J Neurosci Res 2004 76 342 355 15079863 10.1002/jnr.20080

[B50] Smith GST Paez PM Spreuer V Campagnoni CW Boggs JM Campagnoni AT Harauz G Classical 18.5-and 21.5-kDa isoforms of myelin basic protein inhibit calcium influx into oligodendroglial cells, in contrast to golli isoforms J Neurosci Res 2011 89 467 480 21312222 10.1002/jnr.22570 PMC3605075

[B51] Cooper JA Effects of cytochalasin and phalloidin on actin J Cell Biol 1987 105 1473 1478 3312229 10.1083/jcb.105.4.1473 PMC2114638

[B52] Peyrollier K Hajduch E Gray A Litherland GJ Prescott AR Leslie NR Hundal HS A role for the actin cytoskeleton in the hormonal and growth-factor-mediated activation of protein kinase B Biochem J 2000 352 Pt 3 617 622 11104665 PMC1221496

[B53] Kosaras B Kirschner DA Radial component of CNS myelin: junctional subunit structure and supramolecular assembly J Neurocytol 1990 19 187 199 2113569 10.1007/BF01217297

[B54] Karthigasan J Kosaras B Nguyen J Kirschner DA Protein and lipid composition of radial component-enriched CNS myelin J Neurochem 1994 62 1203 1213 8113804 10.1046/j.1471-4159.1994.62031203.x

[B55] Receveur-Bréchot V Bourhis JM Uversky VN Canard B Longhi S Assessing protein disorder and induced folding Proteins 2006 62 24 45 16287116 10.1002/prot.20750

[B56] Arvanitis DN Min W Gong Y Heng YM Boggs JM Two types of detergent-insoluble, glycosphingolipid/cholesterol-rich membrane domains from isolated myelin J Neurochem 2005 94 1696 1710 16045452 10.1111/j.1471-4159.2005.03331.x

[B57] Foster LM Phan T Verity AN Bredesen D Campagnoni AT Generation and analysis of normal and *shiverer* temperature-sensitive immortalized cell lines exhibiting phenotypic characteristics of oligodendrocytes at several stages of differentiation Dev Neurosci 1993 15 100 109 8168435 10.1159/000111322

[B58] Verity AN Bredesen D Vonderscher C Handley VW Campagnoni AT Expression of myelin protein genes and other myelin components in an oligodendrocytic cell line conditionally immortalized with a temperature-sensitive retrovirus J Neurochem 1993 60 577 587 7678286 10.1111/j.1471-4159.1993.tb03188.x

[B59] Smith GST Seymour LV Boggs JM Harauz G The 21.5-kDa isoform of myelin basic protein has a non-traditional PY-nuclear-localization signal Biochem Biophys Res Commun 2012 422 670 675 22609403 10.1016/j.bbrc.2012.05.051 PMC3526657

[B60] Smith GST Samborska B Hawley SP Klaiman JM Gillis TE Jones N Boggs JM Harauz G Nucleus-localized 21.5-kDa myelin basic protein promotes oligodendrocyte proliferation and enhances neurite outgrowth in coculture, unlike the plasma membrane-associated 18.5-kDa isoform J Neurosci Res 2013 91 349 362 23184356 10.1002/jnr.23166 PMC3569502

[B61] Tsai CJ Ma B Nussinov R Protein-protein interaction networks: how can a hub protein bind so many different partners? Trends Biochem Sci 2009 34 594 600 19837592 10.1016/j.tibs.2009.07.007 PMC7292551

[B62] Mackay JP Sunde M Lowry JA Crossley M Matthews JM Protein interactions: is seeing believing? Trends Biochem Sci 2007 32 530 531 17980603 10.1016/j.tibs.2007.09.006

[B63] Wissmueller S Font J Liew CW Cram E Schroeder T Turner J Crossley M Mackay JP Matthews JM Protein-protein interactions: analysis of a false positive GST pulldown result Proteins 2011 79 2365 2371 21638332 10.1002/prot.23068

[B64] Kutzera J Hoefsloot HC Malovannaya A Smit AB Van MI Smilde AK Inferring protein inverted question markprotein interaction complexes from immunoprecipitation data BMC Res Notes 2013 6 468 24237943 10.1186/1756-0500-6-468 PMC3874675

[B65] DeBruin LS Haines JD Wellhauser LA Radeva G Schonmann V Bienzle D Harauz G Developmental partitioning of myelin basic protein into membrane microdomains J Neurosci Res 2005 80 211 225 15772981 10.1002/jnr.20452

[B66] DeBruin LS Haines JD Bienzle D Harauz G Partitioning of myelin basic protein into membrane microdomains in a spontaneously demyelinating mouse model for multiple sclerosis Biochem Cell Biol 2006 84 993 1005 Special issue on Membrane Proteins in Health and Disease 17215885 10.1139/o06-180

[B67] Krämer-Albers EM White R From axon-glial signalling to myelination: the integrating role of oligodendroglial Fyn kinase Cell Mol Life Sci 2011 68 2003 2012 21207100 10.1007/s00018-010-0616-z PMC11114493

[B68] Ammer AG Weed SA Cortactin branches out: roles in regulating protrusive actin dynamics Cell Motil Cytoskeleton 2008 65 687 707 18615630 10.1002/cm.20296 PMC2561250

[B69] Fanning AS Jameson BJ Jesaitis LA Anderson JM The tight junction protein ZO-1 establishes a link between the transmembrane protein occludin and the actin cytoskeleton J Biol Chem 1998 273 29745 29753 9792688 10.1074/jbc.273.45.29745

[B70] Li X Ionescu AV Lynn BD Lu S Kamasawa N Morita M Davidson KG Yasumura T Rash JE Nagy JI Connexin47, connexin29 and connexin32 co-expression in oligodendrocytes and Cx47 association with zonula occludens-1 (ZO-1) in mouse brain Neuroscience 2004 126 611 630 15183511 10.1016/j.neuroscience.2004.03.063 PMC1817902

[B71] Penes MC Li X Nagy JI Expression of zonula occludens-1 (ZO-1) and the transcription factor ZO-1-associated nucleic acid-binding protein (ZONAB)-MsY3 in glial cells and colocalization at oligodendrocyte and astrocyte gap junctions in mouse brain Eur J Neurosci 2005 22 404 418 16045494 10.1111/j.1460-9568.2005.04225.x

[B72] Devaux J Gow A Tight junctions potentiate the insulative properties of small CNS myelinated axons J Cell Biol 2008 183 909 921 19047465 10.1083/jcb.200808034 PMC2592840

[B73] Bauer NG Richter-Landsberg C Ffrench-Constant C Role of the oligodendroglial cytoskeleton in differentiation and myelination Glia 2009 57 1691 1705 19455583 10.1002/glia.20885

[B74] Song J Goetz BD Baas PW Duncan ID Cytoskeletal reorganization during the formation of oligodendrocyte processes and branches Mol Cell Neurosci 2001 17 624 636 11312599 10.1006/mcne.2001.0974

[B75] Young KM Psachoulia K Tripathi RB Dunn SJ Cossell L Attwell D Tohyama K Richardson WD Oligodendrocyte dynamics in the healthy adult CNS: evidence for myelin remodeling Neuron 2013 77 873 885 23473318 10.1016/j.neuron.2013.01.006 PMC3842597

[B76] Richter-Landsberg C The cytoskeleton in oligodendrocytes: Microtubule dynamics in health and disease J Mol Neurosci 2008 35 55 63 18058074 10.1007/s12031-007-9017-7

[B77] Tantos A Han KH Tompa P Intrinsic disorder in cell signaling and gene transcription Mol Cell Endocrinol 2012 348 457 465 21782886 10.1016/j.mce.2011.07.015

[B78] Guharoy M Szabo B Martos SC Kosol S Tompa P Intrinsic structural disorder in cytoskeletal proteins Cytoskeleton (Hoboken) 2013 70 550 571 23761374 10.1002/cm.21118

[B79] Cumberworth A Lamour G Babu MM Gsponer J Promiscuity as a functional trait: intrinsically disordered regions as central players of interactomes Biochem J 2013 454 361 369 23988124 10.1042/BJ20130545

[B80] Uversky VN Dunker AK The case for intrinsically disordered proteins playing contributory roles in molecular recognition without a stable 3D structure F1000 Biol Rep 2013 5 1 23361308 10.3410/B5-1 PMC3542772

[B81] Habchi J Tompa P Longhi S Uversky VN Introducing protein intrinsic disorder Chem Rev 2014 in press online (dx.doi.org/10.1021/cr400514h) 10.1021/cr400514h 24739139

