# Supplementary material for: Interaction of myelin basic protein with cytoskeletal and signaling proteins in cultured primary oligodendrocytes and N19 oligodendroglial cells
Source: BMC Res Notes. 2014 Jun 24;7:387. doi: 10.1186/1756-0500-7-387 (PMC4078013; doi:10.1186/1756-0500-7-387)
Supplement: Additional file 1 — The supplementary files available in the online version of this paper comprise Figure S1 (flowchart of experimental procedure) and Tables S1-S4 (interpretations of Western blots in Figures 1–4, respectively). [file 1756-0500-7-387-S1.pdf]

Supplementary Information

Interaction of Myelin Basic Protein with Cytoskeletal and Signaling Proteins in Cultured Primary Oligodendrocytes and N19 Oligodendroglial Cells

Joan M. Boggs[1,2,*],
Lopamudra Homchaudhuri[1],
Godha Ranagaraj[1],
Yuanfang Liu[1,4]
Graham S. T. Smith[3], and
George Harauz[3]

[1]Molecular Structure and Function Program, Research Institute, Hospital for Sick Children, Toronto, Ontario, Canada;

[2]Department of Laboratory Medicine and Pathobiology, University of Toronto, Toronto, Ontario Canada;

[3]Department of Molecular and Cellular Biology, University of Guelph, Guelph, Ontario, Canada

[4]Present address: Neurosciences and Mental Health Program, Research Institute, Hospital for Sick Children, Toronto, Ontario, Canada.

[*]To whom correspondence should be addressed: Dr. Joan M. Boggs, Molecular Structure and Function Program, Peter Gilgan Centre for Research and Learning, Research Institute, Hospital for Sick Children, 686 Bay St., Toronto, Ontario, Canada M5G 0A4. Phone: 416-813-5919; Fax: 416-813-5022; Email: jmboggs@sickkids.ca.

[*]To whom correspondence should be addressed: Dr. George Harauz, Molecular and Cellular Biology, University of Guelph, 50 Stone Road East, Guelph, Ontario, N1G 2W1, Canada. Phone: 519-824-4120-52535; FAX: 519-837-1802; Email: gharauz@uoguelph.ca.

Supplementary Figure S1. Flowchart of procedures used for cell extraction, immunoprecipitation, and western blotting.

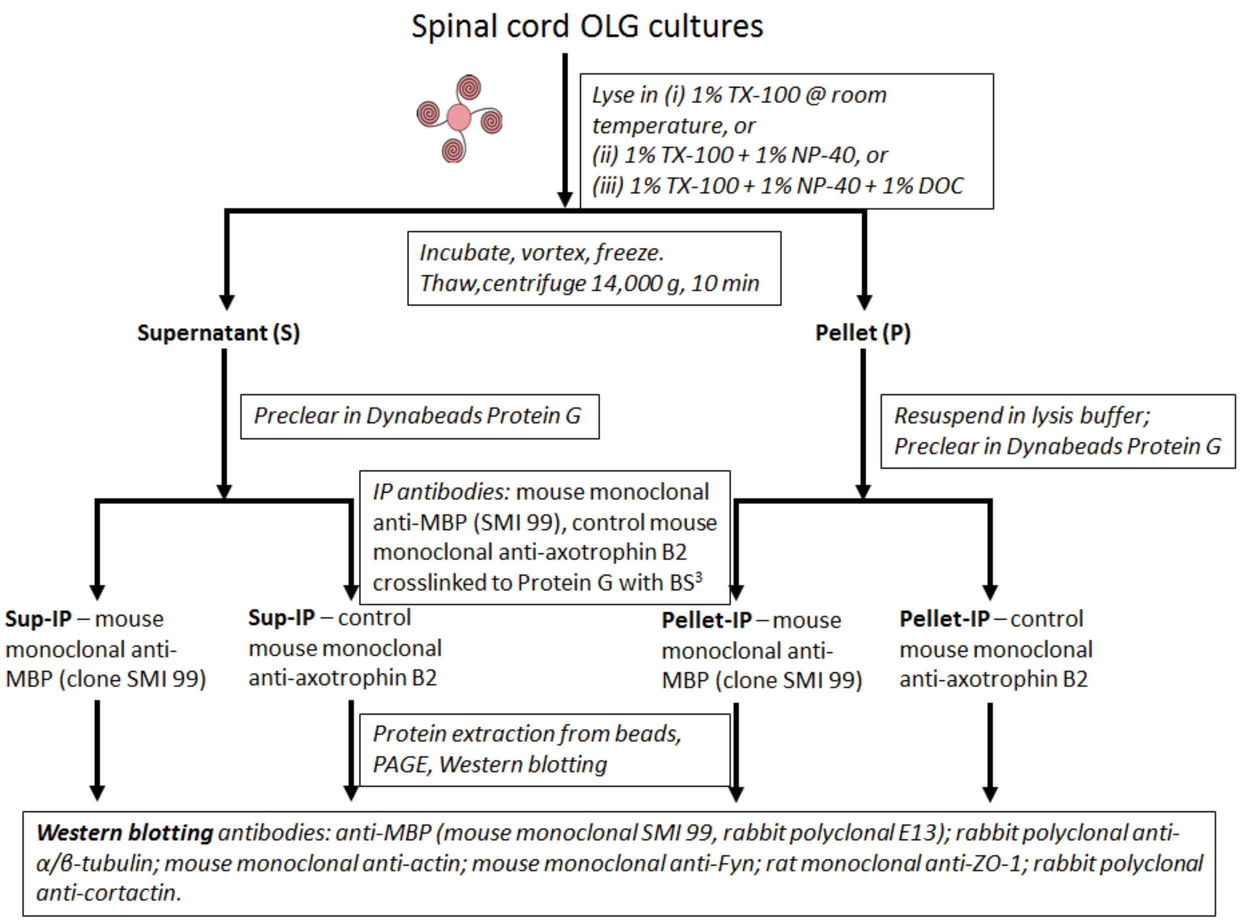

Supplementary Table S1. Interpretation of **Figure 1** – Western blot of primary OLGs extracted with buffer containing 1% TX-100. Samples were immunoprecipitated with anti-MBP (SMI 99) or control Abs, and detected using rabbit polyclonal anti-MBP (E13), mouse monoclonal anti-MBP (SMI 99), rabbit polyclonal anti-tubulin, and mouse monoclonal anti-actin antibodies.

(Abbreviations: "n.d." = "not done"; "-" = "*nil*"; l.c. = IgG light chain; h.c. = IgG heavy chain.)

Figure-Lane	Description	Blotting 1°-Ab	Detection	Interpretation
F1-L1	Supernatant	MBP E13	3 bands, 16, 24, 27 kDa	14-, 18.5-, 21.5-kDa MBP isoforms
		MBP SMI 99	2 bands, 24, 27 kDa	18.5-, 21.5-kDa MBP isoforms
		Tubulin	1 band, 55 kDa	Tubulin
		Actin	1 band, 45 kDa (see Fig. 2a,b)	Actin
F1-L2	Sup-IP anti-MBP SMI 99	MBP E13	3 bands, 16, 24, 27 kDa;<br><br>1band, 30 kDa	14-, 18.5-, 21.5-kDa MBP isoforms;<br>IgG l.c. (after reaction with BS^3)
		MBP SMI 99	2 bands, 24, 27 kDa;<br><br>1 band, 30 kDa	18.5-, 21.5-kDa MBP isoforms;<br>IgG l.c. (after reaction with BS^3)
		Tubulin	-	
		Actin	1 band, 45 kDa	Actin
F1-L3	Sup-IP control	MBP E13	3 bands, 16, 24, 27 kDa;<br><br>1 band, 30 kDa	14-, 18.5-, 21.5-kDa MBP isoforms;<br>IgG l.c. (after reaction with BS^3)
		MBP SMI 99	2 bands, 24, 27 kDa;<br><br>1 band, 30 kDa	18.5-, 21.5-kDa MBP isoforms;<br>IgG l.c. (after reaction with BS^3)
		Tubulin	-	
		Actin	1 band, 45 kDa	Actin
F1-L4	Pellet	MBP E13	3 weak bands, 16, 24, 27 kDa	14-, 18.5-, 21.5-kDa isoforms of MBP
		MBP SMI 99	2 bands, 24, 27 kDa	18.5-, 21.5-kDa MBP isoforms

		Tubulin	1 band, 55 kDa	Tubulin
		Actin	1 band, 45 kDa	Actin
F1-L5	Pellet-IP anti-MBP SMI 99	MBP E13	3 bands, 16, 24, 27 kDa;<br><br>1 band, 30 kDa	14-, 18.5-, 21.5-kDa MBP isoforms;<br>IgG l.c. (after reaction with BS^3)
		MBP SMI 99	2 bands, 24, 27 kDa;<br><br>1 band, 30 kDa	18.5-, 21.5-kDa MBP isoforms;<br>IgG l.c. (after reaction with BS^3)
		Tubulin	1 band, 55 kDa	Tubulin
		Actin	1 band, 45 kDa	Actin
F1-L6	Pellet-IP control	MBP E13	4 weak bands, 16, 24, 27, 30 kDa	14-, 18.5-, 21.5-kDa MBP isoforms; IgG l.c. (reacted with BS^3)
		MBP SMI 99	3 bands, 24, 27, 30 kDa	18.5-, 21.5-kDa MBP isoforms; IgG l.c. (reacted with BS^3)
		Tubulin	-	
		Actin	1 band, 45 kDa	Actin
F1-L7	IgG anti-MBP SMI 99	MBP E13	-	
		MBP SMI 99	-	
		Tubulin	-	
		Actin	1 band, 55 kDa	IgG h.c.
F1-L8	IgG control	MBP E13	-	
		MBP SMI 99	1 band, 28 kDa	IgG l.c.
		Tubulin	-	
		Actin	1 band, 55 kDa	IgG h.c.

Supplementary Table S2. Interpretation of **Figure 2(a,b)** – Western blot of primary OLGs extracted with buffer containing 1% TX-100 plus 1% NP40. Samples were immunoprecipitated with anti-MBP (SMi 99) or a control Ab, and detected using rabbit polyclonal anti-MBP (E13), rabbit polyclonal anti-tubulin, mouse monoclonal anti-actin, rat monoclonal anti-ZO-1, and mouse monoclonal anti-Fyn antibodies.

(Abbreviations: "n.d." = "not done"; "-" = "*nil*"; l.c. = IgG light chain; h.c. = IgG heavy chain; 1°-Ab = primary antibody.)

**Figure-Lane**	**Description**	**Blotting 1°-Ab**	**Detection**	**Interpretation**
F2-L1	Standard	MBP E13	1 band, 24 kDa	18.5-kDa MBP
		Tubulin	1 band, 55 kDa	Tubulin
		Actin	1 band, 45 kDa	Actin
		ZO-1	n.d.	
		Fyn	n.d.	
F2-L2	Supernatant	MBP E13	4 bands, 16, 23, 24, 27 kDa	14-, 17-, 18.5-, 21.5-MBP isoforms
		Tubulin	1 band, 55 kDa	Tubulin
		Actin	1 band, 45 kDa	Actin
		ZO-1	several bands, 140-220 kDa	ZO-1 isoforms
		Fyn	1 band, 60 kDa	Fyn
F2-L3	Sup-IP anti-MBP SMI 99	MBP E13	4 bands, 16, 23, 24, 27 kDa	14-, 17-, 18.5-, 21.5-MBP isoforms
		Tubulin	1 band, 55 kDa	Tubulin
		Actin	1 band, 45 kDa	Actin
		ZO-1	several bands, 140-220 kDa	ZO-1 isoforms
		Fyn	1 band, 60 kDa	Fyn
F2-L4	Sup-IP control	MBP	2 bands, 16, 24 kDa	14-, 18.5-kDa MBP isoforms
		Tubulin	1 weak band, 55 kDa	Tubulin
		Actin	1 band, 45 kDa	Actin
		ZO-1	1 band, 220 kDa	ZO-1 isoform
		Fyn	1 weak band, 60 kDa;<br>1 band, 75 kDa	Fyn;<br>crosslinked IgG l.c. + h.c.
F2-L5	Pellet	MBP	3 weak bands, 23, 24, 27 kDa	17-, 18.5-, 21.5-kDa MBP isoforms
		Tubulin	1 band, 55 kDa	Tubulin
		Actin	1 band, 45 kDa	Actin
		ZO-1	1 band, 220 kDa	ZO-1 isoform

		Fyn	1 band, 60 kDa	Fyn
F2-L6	Pellet-IP anti-MBP SMI 99	MBP	4 bands, 16, 23, 24, 27 kDa	14-, 17-, 18.5-, 21.5-kDa MBP isoforms
		Tubulin	1 band, 55 kDa	Tubulin
		Actin	1 band, 45 kDa	Actin
		ZO-1	several bands, 140-220 kDa	ZO-1 isoforms
		Fyn	1 band, 60 kDa	Fyn
F2-L7	Pellet-IP control	MBP	4 bands, 16, 23, 24, 27 kDa	14-, 17-, 18.5-, 21.5-kDa MBP isoforms
		Tubulin	1 band, 55 kDa	Tubulin
		Actin	1 band, 45 kDa	Actin
		ZO-1	1 band, 220 kDa	ZO-1 isoform
		Fyn	1 band, 60 kDa;<br>1 band, 75 kDa	Fyn;<br>crosslinked IgG l.c. + h.c.
F2-L8	IgG anti-MBP SMI 99	MBP	-	
		Tubulin	-	
		Actin	1 band, 55 kDa	IgG h.c.
		ZO-1	-	
		Fyn	1 band, 55 kDa	IgG h.c.
F2-L9	IgG control	MBP	-	
		Tubulin	-	
		Actin	1 band, 55 kDa	IgG h.c.
		ZO-1	-	
		Fyn	1 band, 55 kDa	IgG h.c.

Supplementary Table S3. Interpretation of **Figure 3** – Western blot of primary OLGs extracted with buffer containing 1% TX-100 plus 1% NP40. Samples were immunoprecipitated using mouse monoclonal anti-MBP (SMI 99) or control antibodies, and detected using rabbit polyclonal anti-cortactin antibody.

(Abbreviations: “n.d.” = “not done”; “-“ = “*nil*”.)

**Figure-Lane**	**Description**	**Blotting 1°-Ab**	**Detection**	**Interpretation**
F3-L1	Supernatant	cortactin	2 bands, 78, 82 kDa	cortactin isoforms
F3-L2	Blank	cortactin	-	
F3-L3	Pellet	cortactin	2 bands, 78, 82 kDa	cortactin isoforms
F3-L4	Sup-IP anti-MBP SMI 99	cortactin	2 bands, 78, 82 kDa	cortactin isoforms
F3-L5	Sup-IP control	cortactin	2 weak bands, 78, 82 kDa	cortactin isoforms
F3-L6	Blank	cortactin	-	
F3-L7	Pellet-IP anti-MBP SMI 99	cortactin	2 bands, 78, 82 kDa	cortactin isoforms
F3-L8	Pellet-IP control	cortactin	2 bands, 78, 82 kDa	cortactin isoforms
F3-L9	IgG anti-MBP SMI 99	cortactin	-	
F3-L10	IgG control		-	

Supplementary Table S4. Interpretation of **Figure 4** – Western blot of primary OLGs extracted with buffer containing 1% TX-100 plus 1% NP40 plus 1% DOC. Samples were immunoprecipitated with monoclonal anti-MBP (SMI 99) or control antibodies, and detected using rabbit polyclonal anti-MBP (E13), rabbit polyclonal anti-tubulin, mouse monoclonal anti-actin, and rat monoclonal anti-ZO-1 antibodies.

(Abbreviations: "n.d." = "not done"; "-" = "*nil*".)

**Figure-Lane**	**Description**	**Blotting 1°-Ab**	**Detection**	**Interpretation**
F4-L1	Standard	MBP	1 band, 24 kDa	18.5 kDa MBP isoform
		Tubulin	1 band, 55 kDa	Tubulin
		Actin	1 band, 45 kDa (see Fig. 2)	Actin
		ZO-1	n.d.	
F4-L2	Supernatant	MBP	3 bands, 16, 24, 27 kDa	14, 18.5, 21.5 kDa MBP isoforms
		Tubulin	1 heavy band, 55 kDa	Tubulin
		Actin	1 heavy band, 45 kDa	Actin
		ZO-1	3 bands, 140-220 kDa	ZO-1 isoforms
F4-L3	Sup-IP anti-MBP SMI 99	MBP	2 bands, 24, 27 kDa;<br><br>1 band 30 kDa	18.5, 21.5 kDa MBP isoforms;<br>IgG l.c. (after reaction with BS^3)
		Tubulin	-	
		Actin	1 weak band, 45 kDa	Actin
		ZO-1	-	
F4-L4	Sup-IP control	MBP	1 band, 30 kDa	IgG l.c. (after reaction with BS^3)
		Tubulin	-	
		Actin	1 weak band, 45 kDa	Actin
		ZO-1	-	
F4-L5	Pellet	MBP	3 weak bands, 16, 24, 27 kDa	14, 18.5, 21.5 kDa MBP isoforms
		Tubulin	1 band, 55 kDa	Tubulin
		Actin	1 band, 45 kDa	Actin
		ZO-1	-	
F4-L6	Pellet-IP anti-MBP SMI 99	MBP	1 band, 30 kDa	IgG l.c. (after reaction with BS^3)

		Tubulin	-	
		Actin	-	
		ZO-1	-	
F4-L7	Pellet-IP control	MBP	1 band, 30 kDa	IgG l.c. (after reaction with BS^3)
		Tubulin	-	
		Actin	-	
		ZO-1	-	
F4-L8	IgG anti-MBP SMI 99	MBP	-	
		Tubulin	-	
		Actin	-	
		ZO-1	-	
F4-L9	IgG control	MBP	-	
		Tubulin	-	
		Actin	-	
		ZO-1	-	
